# Verification of the delivered patient radiation dose for non‐coplanar beam therapy

**DOI:** 10.1002/acm2.13280

**Published:** 2021-05-22

**Authors:** Ivan Kutuzov, Timothy Van Beek, Boyd M.C. McCurdy

**Affiliations:** ^1^ Department of Physics and Astronomy University of Manitoba Winnipeg Manitoba Canada; ^2^ Medical Physics Department CancerCare Manitoba Winnipeg Manitoba Canada; ^3^ Department of Radiology University of Manitoba Winnipeg Manitoba Canada

**Keywords:** EPID dosimetry, *in vivo* dosimetry, non‐coplanar treatment, QA in radiotherapy, SBRT

## Abstract

**Purpose:**

There is an increased interest in using non‐coplanar beams for radiotherapy, including SBRT and SRS. This approach can significantly reduce doses to organs‐at‐risk, however, it requires stringent quality assurance, especially when a dynamic treatment couch is used. In this work, new functionality that allows using non‐coplanar beam arrangements in addition to conventional coplanar beams was added and validated to the previously developed in vivo dose verification system.

**Methods:**

The existing program code was modified to manage the additional treatment couch parameters: angle and positions. Ten non‐coplanar test plans that use a static couch were created in the treatment planning system. Also, two plans that use a dynamic treatment couch were created and delivered using Varian Developer mode, since the treatment planning system does not support a dynamic couch. All non‐coplanar test trajectories were delivered on a simple geometric phantom, using an Edge linear accelerator (Varian Medical Systems) with the megavoltage imager deployed and acquiring megavoltage transmission images that were used to calculate the delivered 3D dose distributions in the phantom with the updated dose calculation algorithm. The reconstructed dose distributions were compared using the 3D chi‐comparison test with 2%/2mm tolerances to the corresponding reference dose distributions obtained from the treatment planning system.

**Results:**

The chi‐comparison test resulted in at least a 97.0% pass rate over the entire 3D volume for all tested trajectories. For static gantry, static couch non‐coplanar fields, and non‐coplanar arcs using dynamic couch the pass rates observed were at least 98%, while for the static couch, non‐transverse coplanar arc fields, pass rates were at least 97%.

**Conclusions:**

A model‐based 3D dose calculation algorithm has been extended and validated for a variety of non‐coplanar beam trajectories of different complexities. This system can potentially be applied for quality assurance of treatment delivery systems that use complex, non‐coplanar beam arrangements.

## 1 Introduction

Dose escalation and hypofractionation—critical features of Stereotactic body radiation therapy (SBRT) and Stereotactic radiosurgery (SRS)—were made possible in the past by the use of treatment techniques that create a high dose gradient around the planned treatment volume.^1^ The use of non‐coplanar fields to create even more highly conformal dose distributions has been actively investigated especially over the last decade.^2^, ^3^, ^4^, ^5^, ^6^, ^7^, ^8^, ^9^, ^10^, ^11^, ^12^ Non‐coplanar treatment techniques may use a static‐gantry Intensity‐modulated radiation therapy (IMRT) approach (e.g., “4π treatment”) or a dynamic gantry (e.g., RapidArc^TM^) delivery with non‐transverse couch angle. Non‐coplanar Volumetric Modulated Arc Therapy (VMAT) may also employ a dynamic couch to obtain an optimal dose distribution and further reduce the delivery time compared to static beams.

Rwigema et al.^4^ found that the use of 4π static field treatment for head‐and‐neck cancer may potentially reduce mean and maximum doses to various organs‐at‐risks (OARs), such as ipsilateral carotid and contralateral lens, compared to coplanar SBRT techniques. They observed a reduction of the 50% dose spillage volume by 33% when using non‐coplanar beams.^5^ Dong et al. investigated non‐coplanar static couch SBRT for liver^2^ and centrally located lung tumors.^3^ They reported a 51%–70% mean or maximum dose reduction to various abdominal organs and the spinal cord,^2^ and a 32%–72% maximum dose reduction to mediastinal organs.^3^ A recent review article^13^ summarizes the increase in a therapeutic ratio that non‐coplanar radiotherapy approaches can attain, and also draws attention to the importance of prioritizing the development of this group of delivery methods.

Non‐coplanar dynamic VMAT delivery using couch rotation has also been studied. Smyth et al. evaluated the potential of this technique for breast, brain, and prostate treatments.^8^ They reported a significant dose reduction to heart, eye lens, and rectum.^8^ Separate studies of brain tumors by Smyth et al.^7^ and Yang et al.^11^ have also shown noticeable dosimetric improvements using non‐coplanar dose delivery. Popescu et al.^6^ and Fahimian et al.^5^ found significant potential for better target dose conformity using non‐coplanar beams for breast cancer treatment. The recent introduction of HyperArc^TM^ by Varian Medical Systems has made the clinical implementation of VMAT non‐coplanar treatment more accessible. The first results for brain metastases treatment showed a 99% control rate with no acute or late toxicities.^12^ In general, non‐coplanar delivery techniques have been shown to provide dosimetric benefits and are the focus of commercialization efforts, so these options will be seen more frequently in the clinic in the future.

The use of SRS and SBRT techniques constitutes an increased patient safety risk due to the higher doses and fewer fractions used.^1^, ^14^ In the case of wrong dose delivery, the consequences will likely be more serious compared to conventional techniques. Advanced quality assurance (QA) and dose verification procedures help reduce these risks. One of the approaches to accomplish this is the use of in vivo dose verification using megavoltage (MV) transmission images gathered during treatment by an Electronic Portal Imaging Device (EPID), which has been demonstrated to be a powerful tool for error detection in radiotherapy.^15^, ^16^, ^17^, ^18^, ^19^ Previously, CancerCare Manitoba has implemented a 3D in vivo patient dose verification system that relies on transmission EPID images.^20^, ^21^, ^22^, ^23^, ^24^, ^25^ It supports modern radiotherapy techniques that use conventional co‐planar beam arrangements, such as IMRT and VMAT,^23^ and has been validated for those treatment delivery methods using coplanar geometry and static couch at 0°. McCowan et al.^22^ validated the patient dose reconstruction algorithm specifically for the SBRT‐VMAT treatment of prostate, lung, and spinal tumors using a 6X‐SRS beam but also with coplanar beam geometry and static couch at 0°.

To date, our local in vivo patient dose verification system has not supported non‐coplanar treatment fields. However, given the rapid development of non‐coplanar treatment techniques, it is critical to extending the system to include the possibility of non‐coplanar beam arrangements. This would make it possible to verify patient doses delivered using such techniques as non‐coplanar IMRT, non‐coplanar VMAT, and HyperArc^TM^, and potentially strengthen quality assurance measures when those techniques become used regularly. The purpose of this research was to add and validate this new functionality to extend the in vivo patient dose verification system to make it capable of processing non‐coplanar treatment beams.

## 2 METHODS

### 2.1 Linear accelerator and EPID

An EDGE radiosurgery linear accelerator (Varian Medical Systems, Palo Alto, CA) with a flattened 6MV beam and dose rate of 600 MU/min was used for this work. Non‐coplanar plans described in more detail below were delivered to a rectangular solid water IMRT QA phantom (CIVCO, Orange City, IA) used in our clinic for SBRT pre‐treatment QA purposes.

All measurements were performed using linac’s a‐Si 1200 EPID imager. Its active imager area consists of 1280 × 1280 pixels and has dimensions 43 × 43 cm^2^, corresponding to approximately 0.34 mm per pixel. If operated in continuous (cine) mode, all pixels are engaged. In integrated (dosimetry) mode, only 1190 × 1190 pixels are used, corresponding to approximately a 40 × 40 cm^2^ active area. No additional build‐up was used on the EPID.

For static gantry (IMRT) delivery, EPID images were collected using the integrated mode, while for dynamic gantry (VMAT) delivery, continuous acquisition mode was used. Frame averaging was not used, to ensure the highest possible dose calculation accuracy.^21^, ^24^ Varian Developer mode was used to deliver trajectories that involved dynamic couch rotation. To ensure that every EPID frame gets captured, special firmware named frame grabber was used for those trajectories. A frame grabber is an electronic device that captures and saves all individual, digital frames from a video stream (e.g., EPID images sequence). Frame grabber Solios SOL EV‐CL (Matrox Electronic Systems Ltd, Dorval, QC, Canada) was used in this study to collect every EPID frame in continuous imaging mode since the clinical software does not allow raw continuous mode images to be saved to disk. For all deliveries, a source‐to‐detector distance (SDD) of 180 cm was used instead of the more typical 140–150 cm, to avoid gantry‐couch and EPID‐couch collisions and allow maximum freedom in choosing non‐coplanar beam orientations from the entire 4π space surrounding the phantom.

### 2.2 Dose reconstruction algorithm and its modification to handle non‐coplanar beams

A comprehensive radiation transport model previously developed at CancerCare Manitoba has two important functional modes: “forward” calculation and “backprojection.” The forward model is able to simulate photon energy fluence created in the linear accelerator, transport it through the patient/phantom, and convert it to dose in the EPID in order to create a predicted EPID transmission image. In the model, the 6MV beam energy spectrum is divided into 15 discrete energy bins: 0.1, 0.2, 0.3, 0.4, 0.5, 0.6, 0.8, 1.0, 1.25, 1.5, 2.0, 3.0, 4.0, 5.0, and 6.0 MV. The increased sampling in the low energy region is performed to help more accurately account for the EPID energy overresponse (compared to water) at lower energies.^26^ Both focal and extra‐focal primary fluence components for each energy bin are attenuated through the patient/phantom representation (i.e. Computed tomography (CT) volume) using the “Equivalent Homogeneous Phantom” (EHP) approach and also accounting for the inverse‐square law. Then the model uses a superposition of pre‐calculated patient scatter kernels to compute the patient‐generated scatter fluence entering the EPID plane. A library of radially symmetric patient scatter kernels was generated using Monte Carlo simulation of a 6 MV photon pencil beam incident on a series of water slabs of a variety of thicknesses. This forward model also accounts for the optical glare in the phosphor layer of the EPID. The primary, scattered, and backscattered fluences are combined to calculate the total incident EPID fluence.^20^, ^25^ Dose to the EPID’s phosphor layer is calculated using a convolution of the total EPID fluence for each energy bin with the radially symmetric, Monte Carlo simulated EPID dose deposition kernels, which are valid for the a‐Si 1200 EPID used in this work.

The backprojection model analyzes the measured transmission EPID image, removes the patient scatter component, then estimates the primary focal fluence distribution reaching the EPID.^20^, ^23^ The estimate of the corrected primary focal fluence is backprojected to the plane above the patient, using the EHP approach and accounts for exponential attenuation and inverse square effect along each rayline, and then combines with the predicted primary extra‐focal fluence to get an accurate estimate of total fluence incident on the patient. Finally, the patient dose is calculated using an in‐house developed version of the collapsed‐cone convolution (CCC) algorithm originally described by Ahnesjo.^27^ The reconstructed dose distribution is compared with the treatment planning system (TPS) dose distribution using χ‐comparison^28^ to reveal discrepancies in the dose delivery.

**Fig. 1:**
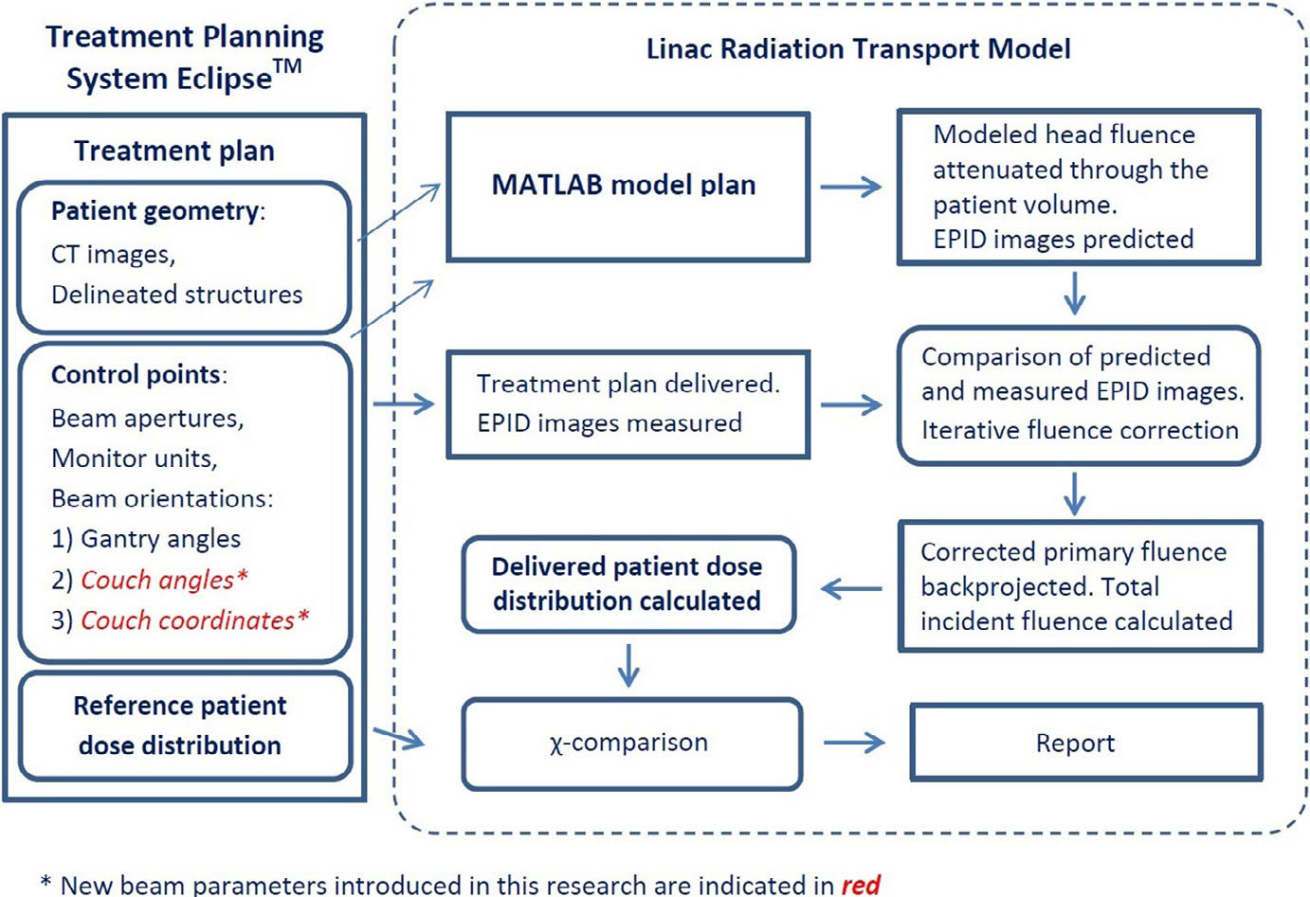
Workflow of the CancerCare Manitoba Linac Model extended to include dynamic couch angle and position.

Prior to the current study, the radiation transport model described above could only work with coplanar Linac trajectories, assuming a static couch angle (default of zero degrees) at all times. The key modification made in this paper is the ability to handle a variable couch angle and couch position. The couch coordinates are obtained from the control point information in the treatment plan. Each control point contains relevant beam delivery parameters: jaw and multileaf collimator (MLC) positions, the number of monitor units (MU) to be delivered, couch position, and the gantry angle. Changing the patient position due to the couch rotation was modeled as a rotation of the patient volume about the vertical couch axis. Introduction of a variable couch angle enabled the model to work with treatment plans that use non‐coplanar beam arrangements. Figure 1 shows the general workflow of the model, including the new couch coordinates.

### 2.3 Test trajectories and collision zones in gantry‐couch coordinates

Non‐coplanar beam treatment geometries can be visualized by depicting the treatment trajectories in the two‐dimensional gantry‐couch coordinate system.^7^, ^8^, ^11^ Before planning the test trajectories, the collision zone map was manually determined for the phantom used for experiments in this work. The phantom was positioned on the treatment couch isocentrically. The couch was positioned at one extreme of its rotation range and then moved in two‐degree increments across its total range. A point of potential collision was defined and recorded if either one of the following conditions was true: the collision prevention system of the Edge started to signal, or the gantry or EPID traveled to within 5 cm proximity of the couch or phantom (this is the current tolerance applied to clinical setups at our institute). In order to maximize the freedom in choosing beam orientations for non‐coplanar trajectory planning purposes, the largest available SDD 180 cm (instead of standard 140 cm), was used.

### 2.4 Trajectory planning and dose calculation for the static couch plans

**Fig. 2:**
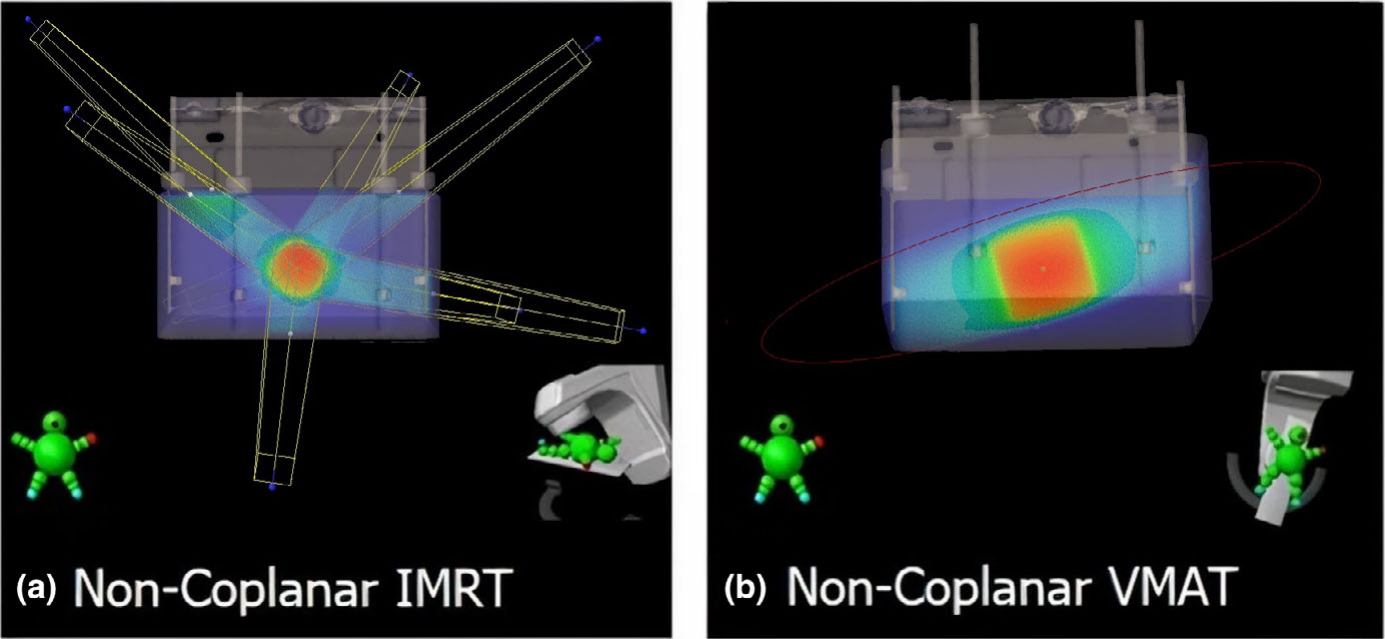
Examples of the test treatment plans that use static couch during beam delivery. (a) Non‐coplanar IMRT. (b) Non‐coplanar VMAT.

In order to validate the correct functionality and logic of the program code modifications, several test treatment plans that involved non‐coplanar beams were created and delivered to the phantom. All of these test plans used simple unmodulated square fields, sized from 5 × 5 cm^2^ to 10 × 10 cm^2^. The beam energy used was 6MV, with a flattening filter. The test plans used for validation were divided into two main categories. Plans in the first category used static couch at a non‐standard angle (or multiple angles for static gantry plans). Plans in the second category used dynamic (rotating) couch during beam delivery. Static couch plans can, in turn, be divided into two sub‐categories:

1. Static couch and multiple static gantry angles, to simulate a non‐coplanar IMRT technique^2^, ^3^, ^4^ [Fig. 2(a)].
2. Static couch (non‐zero angle) with dynamic gantry [Fig. 2(b)]. This approach is sometimes referred to in the literature as a “non‐coplanar” VMAT technique, even though for a single static couch angle, all the beams used in this technique are still coplanar to each other. Recent research has shown that the use of a rotated couch can in some cases result in a dose distribution more conformal to the target, and with better sparing of OARs compared to traditional “co‐planar” VMAT^7^, ^8^ in the patient transverse plane.

​

To test the updated model performance for the 4π treatment (first sub‐category), six plans were created using the following approach. Each plan contained eight static non‐coplanar beams coming from different directions randomly distributed over the entire 4π space around the phantom, except the collision zones. All beams were placed in different octants of space, one beam in each octant; every beam was assigned a weight of 0.125, and the total prescribed dose for each plan was 2 Gy. Square field sizes used varied from 5 × 5 to 10 × 10 cm^2^.

The second category of plans examined used a static couch at a non‐standard angle, combined with a dynamic gantry. Four plans were tested, all using a 10 × 10 cm^2^ open field. The dose prescribed to the isocenter was 2 Gy and delivered using a single 360° full arc in each case. The static couch angles were set to 10° or 15°, either clockwise or counterclockwise. Larger couch angles are not possible to use with full arc delivery due to the risk of potential gantry‐couch collisions. Partial arcs at larger angles were not considered in this test, as their lengths would still be extremely limited due to potential gantry‐couch or EPID‐couch collisions. The parameters used for both types of plans are summarized in Table 1.

**Table 1:** Summary of parameters of the static couch test trajectories used.

Plan #	Plan type	Couch angle	Field size, cm^2^
1	IMRT (static gantry)	Various, 8 unique values per plan	5 × 5
2			6 × 6
3			7 × 7
4			8 × 8
5			9 × 9
6			10 × 10
7	VMAT (dynamic gantry)	10°	10 × 10
8		15°	
9		345° (−15°)	
10		350° (−10°)	

This set of plans was chosen for validation since they represent an incremental increase in complexity between zero couch angle coplanar plans and plans with a fully dynamic couch. Since TPS Eclipse supports a static couch with or without dynamic gantry during beam delivery, the deliverable test plans were created using a conventional planning approach. The dose distributions calculated using the planning system were used by the model as the reference plans for validation (i.e., the experimentally derived in vivo dose distributions were compared to the TPS dose distributions).

### 2.5 Trajectory planning and dose calculation for the dynamic plans

The next level of complexity introduced with the non‐coplanar trajectories tested here is the use of the dynamic treatment couch during beam delivery. Creating these test plans has two challenges:

1. it is not possible to create a deliverable dynamic couch plan using Eclipse, and hence
2. it is not possible to calculate the associated reference dose distributions for the model validation for these plans.

​

These problems stem from the fact that the Eclipse TPS does not currently support dynamic couch for beam delivery or for dose calculation.

To overcome the first problem, the Varian Developer mode was used. Varian Developer (or Research) mode is a special operating mode that enables access to additional advanced control features such as dynamic control including dynamic couch, imaging, and gating capabilities. This mode is not intended for patient treatments. As developer mode is driven by commands written in the XML, the plans of the non‐coplanar trajectories were created as XML scripts. Two dynamic couch trajectories were tested. In the first trajectory, both gantry and couch rotated linearly clockwise from the starting point (181°, 300°) to the ending point (179°, 60°). The figures in the brackets are the gantry and the couch angle. In the second test trajectory, the Linac moved from the initial point (181°, 15°) to the final point (179°, 345°) with the gantry moving clockwise, while the couch moved counterclockwise. A 6MV beam was used for both test trajectories with constant dose rate and MU per gantry/couch angle, and open square fields sized 10 × 10 cm^2^. The monitor units were planned so that the estimated total dose to the isocenter was approximately 2 Gy.

To overcome the second problem of calculating the reference dose distributions, the Eclipse TPS was used in an unconventional way. The entire dynamic trajectory was divided into 120 static beams, uniformly distributed over the couch and gantry angle range. Each beam was assigned an identical number of monitor units to ensure a constant dose rate during beam delivery, keeping the total MU amount the same as in the deliverable XML plans. The total dose was calculated as the sum of dose contributions by each static beam. The total number of beams (i.e., 120) was selected to be close to what is used for clinical VMAT planning (typically 120–180 sampled beams per full 360° arc). This selection provides a relatively small beam angle separation of 3° that helps maintain dose calculation accuracy. Since the treatment planning system is not set up to efficiently calculate dose from hundreds of static beams, the number of beams (120) and beam separation (3°) was chosen as a compromise between the dose calculation accuracy and the computational speed.

All the test treatment plans were delivered using an Edge linear accelerator (Varian Medical Systems) with the EPID deployed and acquiring transmission cine images. Usually, the Record and Verify system of the Linac averages and compresses those images to save server space. However, it results in the loss of valuable dosimetric data, which is why in this experiment a frame grabber, special firmware that allows the user to save every acquired raw EPID frame, was used to prevent loss of dosimetric data.

The acquired cine images were used in this paper for the primary fluence estimate corrections, as described above in II.C, and reconstruction of the dose delivered to the phantom using the updated algorithm. The reconstructed dose distributions were compared against the calculation in the Eclipse TPS, in order to validate the modifications made to the model to extend it to non‐coplanar treatment beams. Chi‐comparison with 2%/2 mm criteria was used to compare dose distributions. Dose comparisons were made using three different sub‐volumes of the phantom: (a) the “body region” corresponds to the entire phantom volume where the dose was calculated, (b) the “infield region” is defined where the TPS dose is greater than 20% but less than 80% of the prescribed dose, and (c) the “high dose region” where the TPS dose is greater than 80% of the prescription dose.

## 3 RESULTS

### 3.1 Map of collision zones

**Fig. 3:**
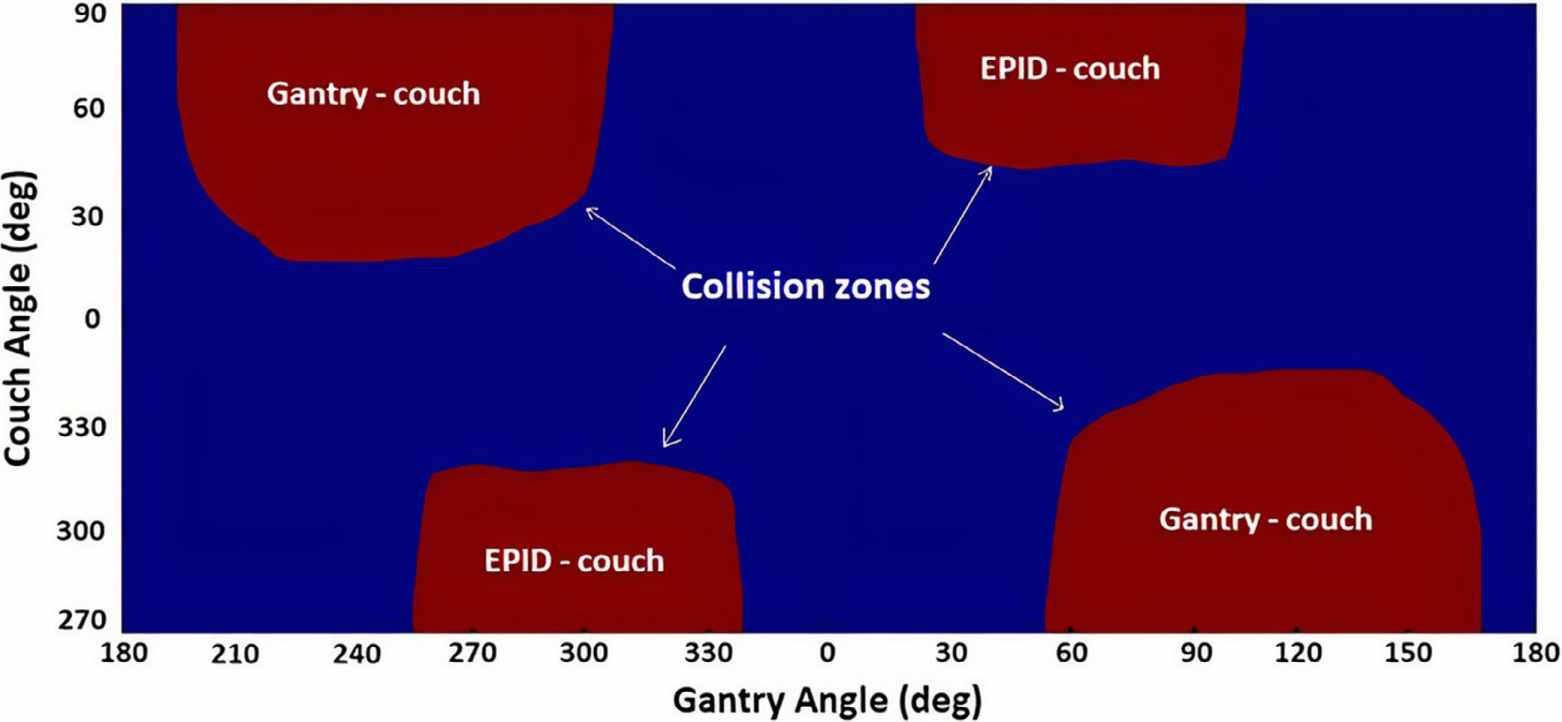
Collision zone map in gantry‐couch coordinates.

The experimentally defined collision zones are shown in Fig. 3. They include possible collisions between gantry and couch, and between EPID and couch. This collision map was used throughout the experiment to ensure the safety of the developed non‐coplanar test trajectories.

### 3.2 Dose reconstruction from static non‐coplanar beams

**Fig. 4:**
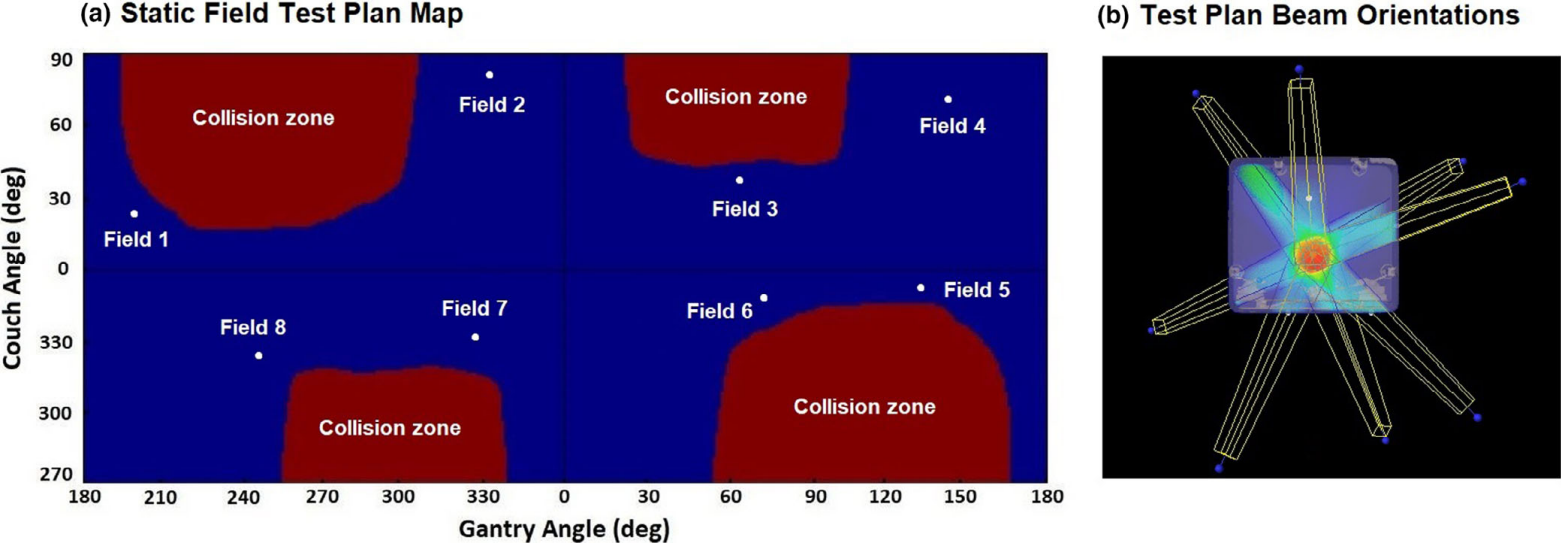
Test plan using eight static, 7 × 7 cm^2^, non‐coplanar fields. (a) Static Field Test Plan Map. (b) Test Plan Beam Orientations.

Figure 4 shows one of the static beam plans used in this experiment. The figure includes the three‐dimensional dose distribution with beam orientations displayed, and the “plan map” which is the overlay of the schematic planned beam representations within the collision zone map.

Table 2 demonstrates the pass rates comparing the transmission EPID reconstructed dose against the reference TPS dose for each test plan that used static, non‐coplanar beams.

**Table 2:** Dose reconstruction results from static fields using χ‐comparison with 2%/2mm criteria.

Plan #	Field size, cm^2^	Body region	Infield region	High dose region
1	5 × 5	98.5%	98.1%	99.3%
2	6 × 6	98.3%	99.9%	98.8%
3	7 × 7	99.0%	99.6%	99.4%
4	8 × 8	98.3%	98.6%	99.8%
5	9 × 9	98.4%	99.0%	99.6%
6	10 × 10	98.7%	99.3%	99.7%

**Fig. 5:**
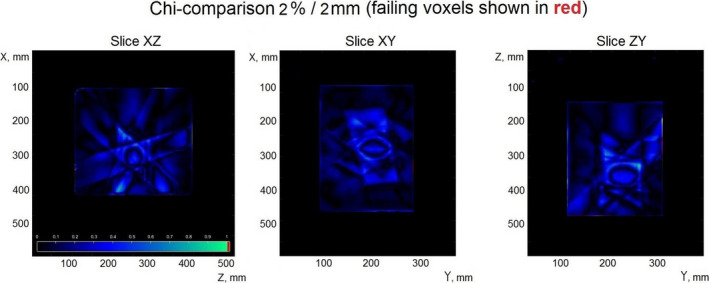
Verification of the dose reconstructed from eight static, non‐coplanar fields (plan #3, Table 2).

Figure 5 shows the results of the χ‐comparison for the same plan (plan number 3 in Table 2). As can be observed, the updated algorithm shows a good comparison in the case of static, non‐coplanar field delivery, with pass rates of at least 98% among the tested plans.

### 3.3 Dose reconstruction from arc fields located in rotated planes

**Fig. 6:**
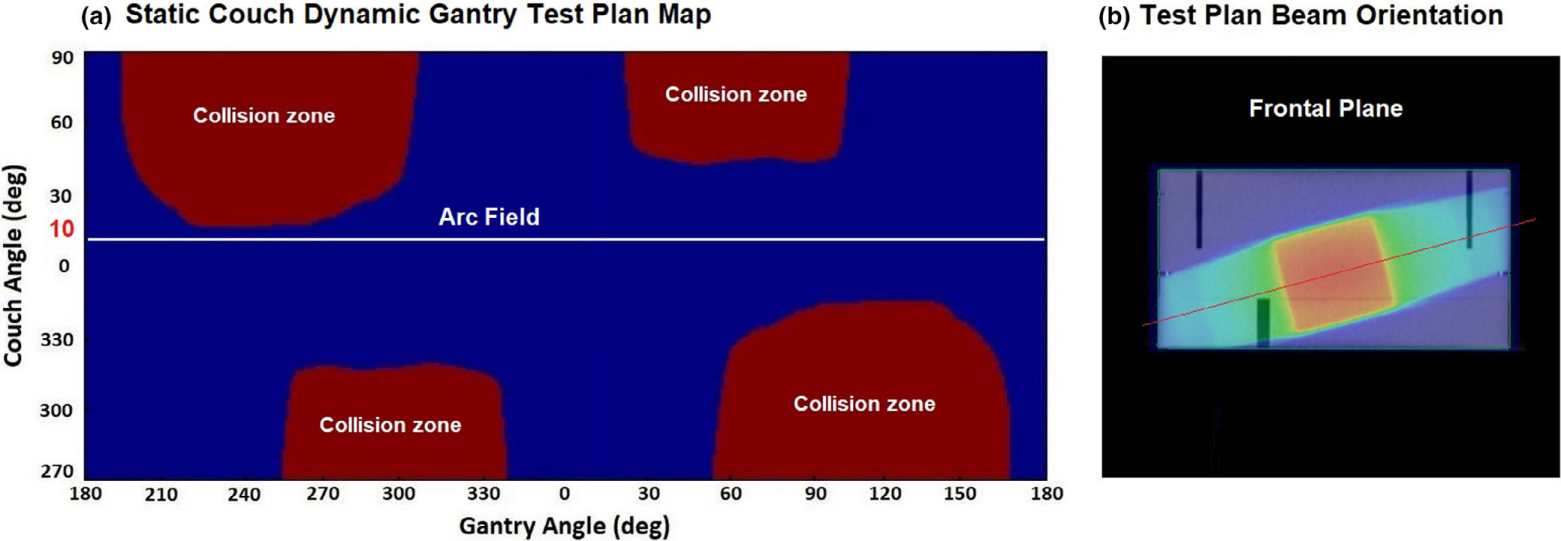
Full arc test plan in the rotated plane (plan #1, Table 3). (a) Static Couch Dynamic Gantry Test Plan Map. (b) Test Plan Beam Orientation.

Figure 6 shows the trajectory of plan #1 from Table 3 and the corresponding treatment plan as it appears in the planning system. It can be seen that the arc plane (couch) is rotated by 10 degrees about the vertical axis through isocenter. The trajectory appears as a horizontal line at a couch angle of 10°. Table 3 demonstrates the pass rates comparing the transmission EPID reconstructed dose against the TPS dose for each test plan that used static, non‐coplanar beams.

**Table 3:** Dose reconstruction from full arcs in rotated planes using χ‐comparison with 2%/2mm criteria.

Plan #	Couch angle	Body region	Infield region	High dose region
1	10°	97.8%	98.4%	97.5%
2	15°	97.1%	97.9%	97.3%
3	345° (−15°)	97.3%	98.0%	97.2%
4	350° (−10°)	97.6%	97.4%	97.8%

**Fig. 7:**
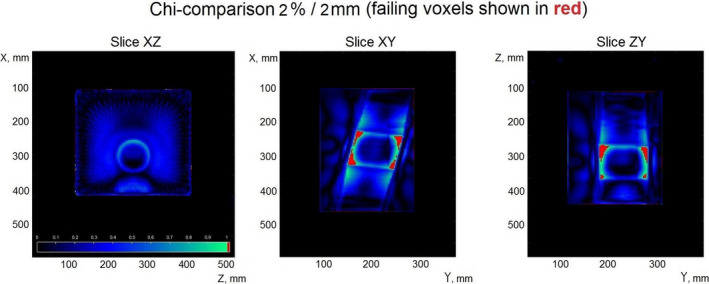
Verification of the dose reconstructed from the full arc in a rotated plane (for plan #1, Table 3).

Figure 7 shows the results of the chi‐comparison of the transmission EPID reconstructed dose against the dose calculated in the Eclipse TPS for plan #1 from Table 3.

The comparison shows some failing voxels just inside the high dose region. These observed dose discrepancies may be due to the high dose gradient in that region which may be more susceptible to small positional uncertainties in the phantom setup. This test configuration in particular has the highest average angle of beam entry with respect to the phantom surface, so this may be a reason for a slightly lower pass rate observed in the phantom body region as compared to the other test scenarios.

### 3.4 Dose reconstruction from non‐coplanar trajectories involving dynamic couch motion

**Fig. 8:**
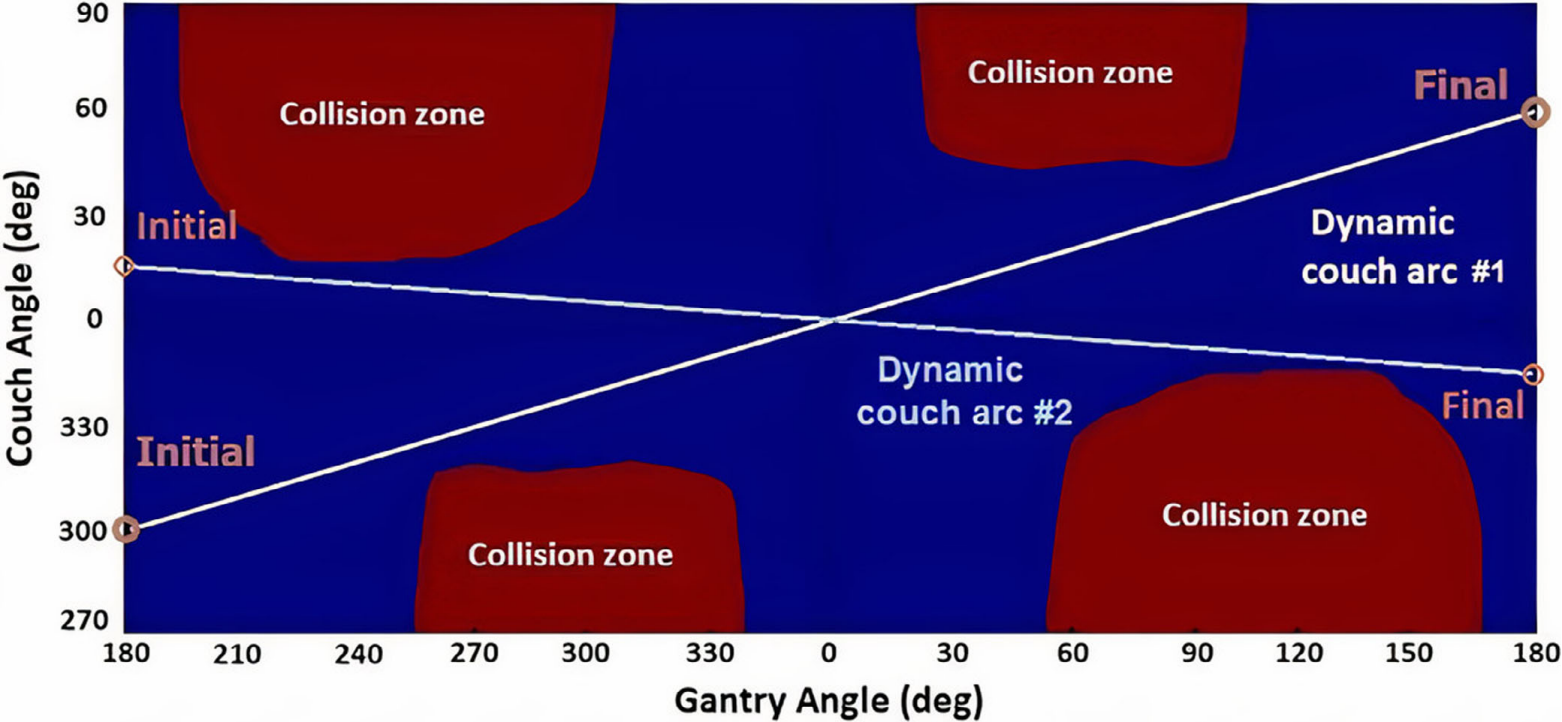
Dynamic couch test plan trajectories.

Figure 8 illustrates both dynamic couch trajectories whose validation results are summarized in Table 4. They are dynamic trajectories with uniform gantry and couch rotation speeds.

**Table 4:** Dose reconstruction results from the dynamic couch test plan using χ‐comparison.

Plan #	Plan coordinates		χ‐comparison pass rate 2%/2mm		
	Initial	Final	Body region	Infield region	High dose region
1	(181°, 300°)	(179°, 60°)	98.2%	98.5%	99.3%
2	(181°, 15°)	(179°, 345°)	98.1%	98.7%	99.2%

**Fig. 9:**
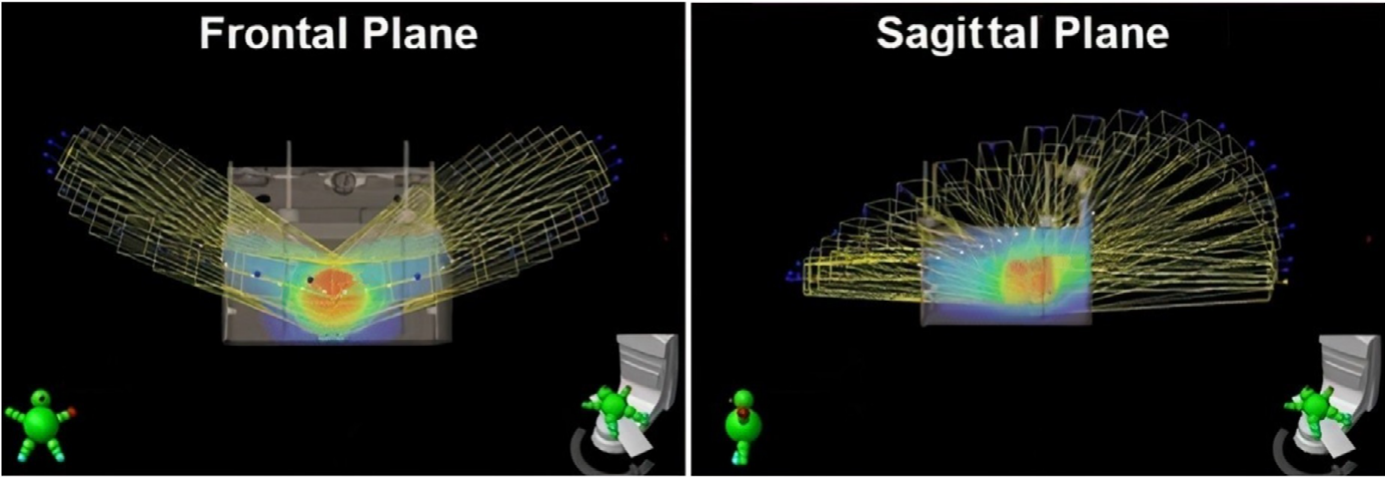
Static beams used for the dose calculation from dynamic couch test plan #1 (of Table 4).

The positions of the beam set that was used for the reference dose calculation in the planning system for plan #1 in this category are shown in Fig. 9. The figure shows the appearance of the beam set, as observed in two different viewing planes—frontal and sagittal—to fully illustrate its non‐coplanar geometry.

Table 4 shows the results of the chi‐comparison of the EPID reconstructed dose against the dose calculated in the Eclipse TPS for the two tested dynamic non‐coplanar trajectories.

**Fig. 10:**
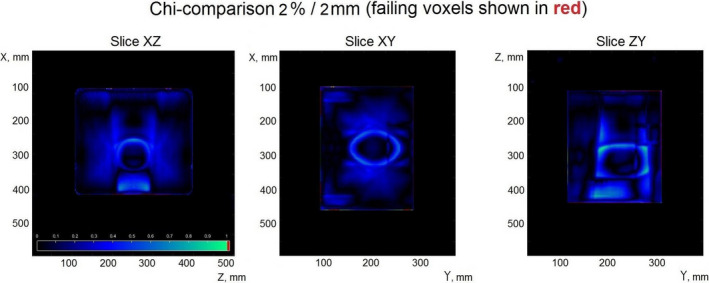
Verification of the dose reconstructed from the dynamic couch test plan #1 (of Table 4).

Figure 10 shows results of the chi‐comparison of the transmission EPID reconstructed dose against the dose calculated in the Eclipse TPS for plan #1 in Table 4.

The tests with the dynamic couch trajectories achieve a pass rate of at least 98% for every dose region, for each tested trajectory.

## 4 DISCUSSION

Currently, there is widespread research aiming to utilize non‐coplanar beam geometry options for radiotherapy delivery, such as the use of many static non‐coplanar beams (known as “4π treatment”), as well as the HyperArc^TM^ technique recently introduced by Varian Medical Systems, based on early development work at McGill University.^29^ Transmission EPID based dosimetry has been long recognized as a powerful patient safety instrument that can be used for in vivo verification of the actual delivered patient radiation dose. Our research group at CancerCare Manitoba has previously developed an in vivo dosimetry method that consists of isolating the primary focal fluence component from the EPID images, back‐projecting it to a plane above the patient, and then using it for the patient dose calculation.

Previously, the CancerCare model only supported co‐planar beam arrangements. However, the purpose of this research was to add non‐coplanar functionality into the existing model by the incorporation of the rotation of the patient/phantom 3D CT data set, to emulate beam orientation changes that occur during non‐coplanar beam delivery. To test and validate the updated dose reconstruction algorithm that now supports non‐coplanar beams, several non‐coplanar test trajectories were created that involved either static or dynamic couch.

The deliverable treatment plans were created using the Eclipse treatment planning system for static couch plans, and the XML scripting language combined with Varian Developer mode (to deliver these plans) for dynamic couch plans. Reference dose calculations were performed using the Eclipse TPS using discretely sampled static beams. The developed test plans were delivered to the geometric solid water QA phantom using an Edge clinical radiosurgery system (Varian Medical Systems). The EPID was deployed during delivery, acquiring megavoltage transmission images that then were used for the dose reconstruction. The frame‐grabber firmware was used to prevent loss of data when saving images in cine mode. The updated in vivo dosimetry algorithm was then used to calculate the dose distribution to the phantom.

The measured dose distributions were compared against the Eclipse reference dose using χ‐comparison with 2%/2mm criteria. For each non‐coplanar plan category, the voxel pass rates obtained were as follows:

1. Static non‐coplanar fields—at least a 98% pass rate among all the tested trajectories,
2. Static couch, dynamic gantry—at least a 97% pass rate among all the tested trajectories,
3. Dynamic couch, dynamic gantry—at least a 98% pass rate among all the tested trajectories.

​

Slightly lower pass rates were observed for the static couch, dynamic gantry plans (“non‐coplanar” VMAT) are likely a result of positional uncertainties in the phantom setup, as this test configuration has the highest angle of beam entry with respect to the phantom surface.

The greatest differences between the reconstructed and the reference dose distributions were observed in the high gradient dose regions corresponding to the geometrical edges of the square fields used. Figure 7 shows failing voxels in those regions for a static couch, dynamic gantry plan. The reasons why χ‐comparison demonstrated increased failure in those regions is likely the high value of the dose gradient and, possibly, misalignment of the phantom with respect to the planned position.

## 5 Conclusion

For all plan types and for all dose regions analyzed, at least a 97% pass rate over the entire 3D dose distribution was observed. A non‐coplanar extension to an existing robust, model‐based 3D dose calculation algorithm was developed that can be used for complex non‐coplanar beam treatment techniques, such as 4π and HyperArc^TM^. The extended model can provide the user with an accurate, three‐dimensional distribution of the actually delivered patient dose. It can potentially be used for both pre‐treatment QA and for in vivo dosimetry.

## Conflict of Interest

The authors declare no conflict of interest.

## Author contributions

Ivan Kutuzov: Made changes in an existing in‐house developed in vivo dose reconstruction model. Created test treatment plans using Eclipse treatment planning system and Varian developer mode. Collected the data ‐ series of transmission megavoltage EPID images used for dose reconstruction. Performed the analysis. Wrote the manuscript.

Timothy Van Beek: Reviewed changes in an existing in‐house developed in vivo dose reconstruction model. Provided overall programming expertise for the model extension. Collected the data ‐ series of transmission megavoltage EPID images used for dose reconstruction. Contributed to analysis tools, specifically chi‐comparison.

Dr. Boyd M.C. McCurdy: Conceived and designed the idea of the experiment. Provided overall guidance and leadership of the experiment through discussions with other authors. Reviewed the results of the experiment. Reviewed the manuscript.

## Data Availability

The data that support the findings of this study are available from the corresponding author upon reasonable request.
